# Is conflict adaptation triggered by feature repetitions? An unexpected finding

**DOI:** 10.3389/fpsyg.2014.01358

**Published:** 2015-02-02

**Authors:** Elke Van Lierde, Kobe Desender, Eva Van den Bussche

**Affiliations:** ^1^Department of Psychology, Vrije Universiteit BrusselBrussels, Belgium; ^2^Department of Experimental-Clinical and Health Psychology, Ghent UniversityGhent, Belgium

**Keywords:** conflict adaptation, Gratton effect, cognitive control, priming, subliminal, feature repetitions

## Abstract

For decades, cognitive adaptation to response conflict has been considered to be the hallmark of cognitive control. Notwithstanding a vast amount of evidence ruling out low-level interpretations of these findings, disbelief still exists with regard to the underlying cause of the observed effects. Especially when considering cognitive adaptation to unconscious conflict, it is still a matter of debate whether repetitions of features between trials might explain this intriguing finding rather than the involvement of unconscious control. To this purpose, we conducted two masked priming experiments in which four different responses to four different stimuli were required. This allowed us to completely eliminate repetitions of prime and target over consecutive trials. Independent of whether conflicting information was presented clearly visible or almost imperceptible, the results showed an unexpected pattern. Contrary to the regular congruency sequence effect (CSE; i.e., classic Gratton effect), in both experiments the congruency effect *increased* following incongruent trials. Interestingly, this reversed effect completely disappeared when we eliminated all trials with feature repetitions from the analysis. A third experiment, in which feature repetitions were excluded *a priori*, showed a small but regular CSE in the error rates only. Given that feature repetitions are theoretically thought to create a regular CSE, our results are not in line with an interpretation in terms of feature repetitions nor with an interpretation in terms of cognitive control. We conclude that examining cognitive adaptation with or without feature repetitions might be more difficult to conceive than is often suggested in the literature.

## INTRODUCTION

In the search for the limits and possibilities of unconscious processing, cognitive control processes have been studied extensively. These processes, which make it possible to behave appropriately in constantly changing environments (e.g., Botvinick et al., 2001), are traditionally thought to require consciousness (Dehaene and Naccache, 2001; Jack and Shallice, 2001). To experimentally test this theoretical assumption, conflict tasks have often been used. In these tasks, participants need to respond to relevant stimulus features while ignoring irrelevant features. For example, in the priming paradigm, participants need to identify a target while ignoring a preceding prime. The relation between the prime and the target is manipulated to create conflict and non-conflict trials. On conflict (also termed incongruent) trials, the prime and target trigger a different response while on non-conflict (also termed congruent) trials both prime and target trigger the same response. Responses are typically slower and error rates higher on incongruent compared to congruent trials (i.e., the congruency effect). Interestingly, participants adapt their behavior after encountering conflicting information, leading to a decrease in the congruency effect. In a seminal study, Gratton et al. (1992) observed that the congruency effect is sharply reduced when the previous trial is incongruent compared to congruent. This congruency sequence effect (CSE) is also called the Gratton effect (Gratton et al., 1992). Although the Gratton effect has been studied the most and is a robust finding in different kinds of conflict tasks, it is just one specific kind of CSE. In theory, also other modulations of the congruency effect can potentially occur, and hence be called CSE. Therefore, in the following, we will refer to the Gratton effect as a ‘regular CSE’ and to all other kinds of sequential modulation of the congruency effect as ‘irregular CSE.’ To explain the occurrence of a *regular* CSE, it is typically assumed that if participants experience conflicting response activations, they try to reduce the influence of the conflicting information on subsequent occasions, by focusing more on the relevant stimulus features and/or ignoring irrelevant features (Botvinick et al., 2001). This leads to conflict adaptation: enhanced performance on incongruent trials and reduced performance on congruent trials, leading overall to a reduced congruency effect.

The regular CSE is traditionally considered to be a result of cognitive control processes (Botvinick et al., 2001), which are assumed to require consciousness (Dehaene and Naccache, 2001; Jack and Shallice, 2001). To get a better grasp on the possibility of unconscious cognitive control, researchers have studied whether this regular CSE still occurs when the conflicting information remains unconscious. Results of this approach, however, are not unambiguous (for a review see Desender and Van den Bussche, 2012; Kunde et al., 2012). While some researchers argue that awareness of the irrelevant information (e.g., the prime) is a prerequisite for this effect to occur (Greenwald et al., 1996; Kunde, 2003; Frings and Wentura, 2008; Ansorge et al., 2011), others have found a reliable regular CSE even when the prime remained unconscious (van Gaal et al., 2010; Desender et al., 2013). These latter findings suggest that unconscious cognitive control is possible. However, recently it has been challenged whether performance on conflict tasks can be used as an index of cognitive control processes at all. Unlike the long-held assumption that the regular CSE reflects cognitive adaptation to conflicting information (i.e., *conflict monitoring account*; Botvinick et al., 2001), it has been argued that this effect can be explained by low-level feature repetitions without the need for control processes (i.e., *feature repetitions accounts*; Mayr et al., 2003; Hommel et al., 2004; Schmidt, 2013). Hitherto, several explanations of the regular CSE in terms of feature repetitions have been proposed. For example, according to Mayr et al. (2003), repetition priming effects (e.g., Pashler and Baylis, 1991) underlie the regular CSE. They pointed out that in a two-alternative forced choice task 50% of II (i.e., an incongruent trial followed by an incongruent trial) and CC (i.e., a congruent trial followed by a congruent trial) trials are complete repetitions. CI (i.e., a congruent trial followed by an incongruent trial) and IC (i.e., an incongruent trial followed by a congruent trial) trials, on the other hand, can never be complete repetitions. Thus, the regular CSE can be explained as a superior performance on II and CC trials due to repetition priming effects. In support of this idea, Mayr et al. (2003) observed a regular CSE when all trials were analyzed but this effect was no longer present after removing all target repetitions (i.e., consecutive trials in which the target is the same) from the analysis. In a similar vein, Hommel et al. (2004) claimed that feature repetitions underlie the regular CSE. They argued that stimulus and response features are combined in an event file (i.e., a common episodic memory representation, Hommel, 1998). Whenever one of these two features is the same as in the previous trial, this event file is reactivated, automatically activating both features on the current trial. This is beneficial when both features are indeed repeated on that trial (i.e., complete S-R repetition). On the other hand, when none of the features is repeated (i.e., complete S-R alternations) no event file will be reactivated, thus no wrong activation needs to be suppressed and reaction times (RTs) will also be fast in those cases. However, if only one of both features repeats (i.e., partial S-R repetition) wrong activation needs to be suppressed, which is detrimental for the performance. Given that II and CC trials are always complete repetitions or complete alternations in two-alternative forced choice tasks, responses will be faster and more accurate on these trials compared to CI and IC trials, which are always partial repetitions. According to Hommel et al. (2004), these differences in performance lead to the regular CSE. Although the gist of the arguments in terms of feature repetitions is identical, the specific details slightly differ between these theories. Hence it is important to rule out all possible sorts of repetitions (i.e., complete as well as partial repetitions).

The debate between both interpretations of the regular CSE (i.e., conflict monitoring account versus feature repetitions accounts) has far-reaching consequences for the broad field of cognitive control, given that the effect is often considered as one of the main expressions of cognitive control. Researchers have to bear the alternative interpretations of the regular CSE in mind when investigating cognitive control. Before any conclusions concerning cognitive control processes can be drawn, confounding influences of feature repetitions need to be ruled out. Therefore, it is also crucial to take these alternative explanations into account when studying the assumption that cognitive control processes require consciousness. In the two studies reporting a reliable regular CSE for unconscious primes (van Gaal et al., 2010; Desender et al., 2013) feature repetitions were not sufficiently controlled for. Given that a two-alternative forced choice task was used, repetition effects could not be fully ruled out (Egner, 2007; Mordkoff, 2012) and the observed effects might not reflect a *pure* conflict adaptation effect. In general, the influence of feature repetitions in conflict tasks is still a large matter of debate. Sometimes the effect vanishes after controlling for the confound of repetitions (e.g., Schmidt and De Houwer, 2011), and sometimes the effect remains present (e.g., Kim and Cho, 2014; Schmidt and Weissman, 2014). Still, in none of these studies the regular CSE was investigated in an unconscious condition. It becomes clear that more research is needed in which feature repetitions are controlled for, especially in the field of consciousness.

In this study, three conflict tasks were set-up to thoroughly test both interpretations of the regular CSE, while simultaneously studying the influence of visibility of the conflicting information. We used a priming paradigm with four stimuli and responses. Using a four-alternative instead of a two-alternative forced choice task enabled us to analyze the regular CSE before and after removing all feature repetitions in a masked and unmasked condition. If we would observe a regular CSE when the primes are masked and if that effect would remain present after controlling for feature repetitions, this would be support for the possibility of unconscious cognitive control. However, if the effect would no longer be present after controlling for this bias, low-level processes (i.e., feature repetitions) instead of cognitive control processes would seem to be the underlying cause of the regular CSE.

## EXPERIMENT 1

Experiment 1 is an extension of previous work (Desender et al., 2013), with the modification that we used four different stimuli and responses instead of two. In Experiment 1A, participants completed a priming task using Arabic numbers as stimuli. In Experiment 1B, participants performed a Stroop priming task with color words as primes (e.g., “yellow”) and colored symbols as targets (e.g., &&&&&; presented in yellow). In these four-alternative forced choice tasks, we could eliminate feature repetitions. This enables us to investigate the contribution of the monitoring of conflict and/or feature repetitions to the regular CSE.

### MATERIALS AND METHODS

#### Participants

Twenty-eight students participated in Experiment 1A. One participant was eliminated because the mean error-rate was above 20% and the mean RT was more than two SDs below the average mean. Another participant was eliminated because of a technical failure. Thus, the final sample of Experiment 1A consisted of 26 participants (23 females), with an age range of 18–27 years (*M* = 19.4, SD = 2.0). 27 students (16 females) participated in Experiment 1B. The participants were between 17 and 22 years old (*M* = 18.9, SD = 1.3).

All participants participated in exchange for course credit and had normal or corrected-to-normal vision. Each signed an informed consent before experimentation.

#### Apparatus and stimuli

Intel Pentium 4 computers with 17-inch LCD screens were used to run the experiment. The refresh rate was set to 60 Hz and stimulus presentation was synchronized with the vertical refresh rate (16.7 ms). For stimulus presentation and data collection E-prime version 1.1. was used. The data were analyzed using SPSS 19. All stimuli were presented on a black background in the center of the screen, using Arial, size 14.

In Experiment 1A, targets were the Arabic numbers “1,” “2,” “8,” and “9.” Primes were the Arabic numbers “1,” “2,” “8,” “9,” and the neutral prime “X.” The forward mask was “#$#” and the backward mask was “$#$.” All stimuli were presented in white. Each prime was combined once with a congruent target and once with an incongruent target^1^. The neutral prime was combined with each possible target. As such, four congruent (11, 99, 22, 88), four incongruent (19, 91, 28, 82) and four neutral prime–target combinations (X1, X9, X2, X8) were created.

In Experiment 1B, targets were strings of five colored ampersands. Four colors were used: yellow, blue, green, and red. The Dutch names of the colors [geel (yellow), blauw (blue), groen (green), and rood (red)] were printed in capital letters in gray and used as primes. In this experiment the neutral prime was “££££.” The forward mask was “#$#$#” and the backward mask was “$#$#$.” Experiment 1B comprised four congruent (YELLOW–yellow, BLUE–blue, GREEN–green, RED–red), four incongruent (YELLOW–blue, BLUE–red, GREEN–yellow, RED–green) and four neutral prime–target combinations (££££-yellow, ££££-blue, ££££-green, ££££-red).

Note that we had two categories of trials (i.e., combinations of 1 and 9 and combinations of 2 and 8) in Experiment 1A while in Experiment 1B the different stimuli were mixed to create incongruent trials.

To fully control for feature repetitions, we consider each trial where the identity of the prime and/or the target is the same as the identity of the prime and/or the target of the previous trial as a repetition (i.e., prime–prime, target–target, prime–target, target–prime).

#### Procedure

In both experiments, all participants completed a practice block, an experimental block and a posttest to assess prime visibility. All these parts were completed once in the masked condition and once in the unmasked condition.

**FIGURE 1:**
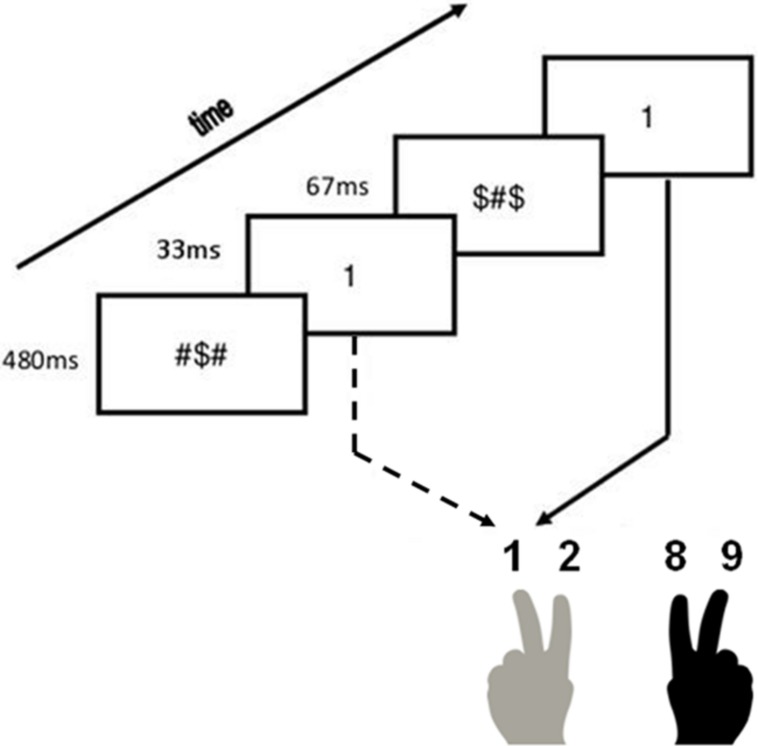
**Experimental procedure.** Example of a congruent trial in the masked condition.

Each trial started with a forward mask presented for 480 ms, followed by a prime for 33 ms. Afterward, a backward mask appeared for 67 ms in the masked condition, or a blank screen in the unmasked condition. Finally, the target was presented until a response was made. These specific timing parameters were chosen because they proved effective in reducing prime visibility in previous research (Desender et al., 2013). Participants needed to categorize the target as quickly and accurately as possible. In Experiment 1A, participants had to press the corresponding numerical key on the top of a standard qwerty keyboard (“1” with the left middle finger, “2” with the left index finger, “8” with the right index finger and “9” with the right middle finger). In Experiment 1B, participants had to respond by pressing the following keys on a qwerty keyboard: “d” with the left middle finger for yellow ampersands, “f” with the left index finger for blue ampersands, “j” with the right index finger for red ampersands and “k” with the right middle finger for green ampersands. Colored stickers were applied on each of these keys to avoid any confusion. The inter-stimulus interval was set to 1000 ms. In **Figure 1**, an example of an experimental trial is shown.

Participants started the masked condition with eight practice trials, during which no prime was shown. Afterward, they were presented with 360 randomly selected experimental trials with an equal amount of congruent, incongruent and neutral trials. After the experimental trials, participants were informed about the presence of the primes and they then completed a posttest where they had to categorize the prime instead of the target. The posttest comprised 120 trials, identical to the experimental trials with the exclusion of neutral trials. Participants were instructed to perform this task at their own pace. Next, participants performed the same three parts in the unmasked condition.

### RESULTS

Reaction times above 1000 ms and below 200 ms (2.1% of the data in Experiment 1A; 4.1% in Experiment 1B), trials on which an error was made (4.6% in Experiment 1A; 4.3% in Experiment 1B) and trials following an error (4.9% in Experiment 1A; 4.7% in Experiment 1B) were excluded from further analysis. Mean RTs of correct trials and mean error rates were submitted to a repeated measures ANOVA with current congruency (two levels: congruent or incongruent)^2^, previous congruency (two levels: congruent or incongruent) and visibility (two levels: unmasked or masked) as within-subject factors.

#### Reaction times

**FIGURE 2:**
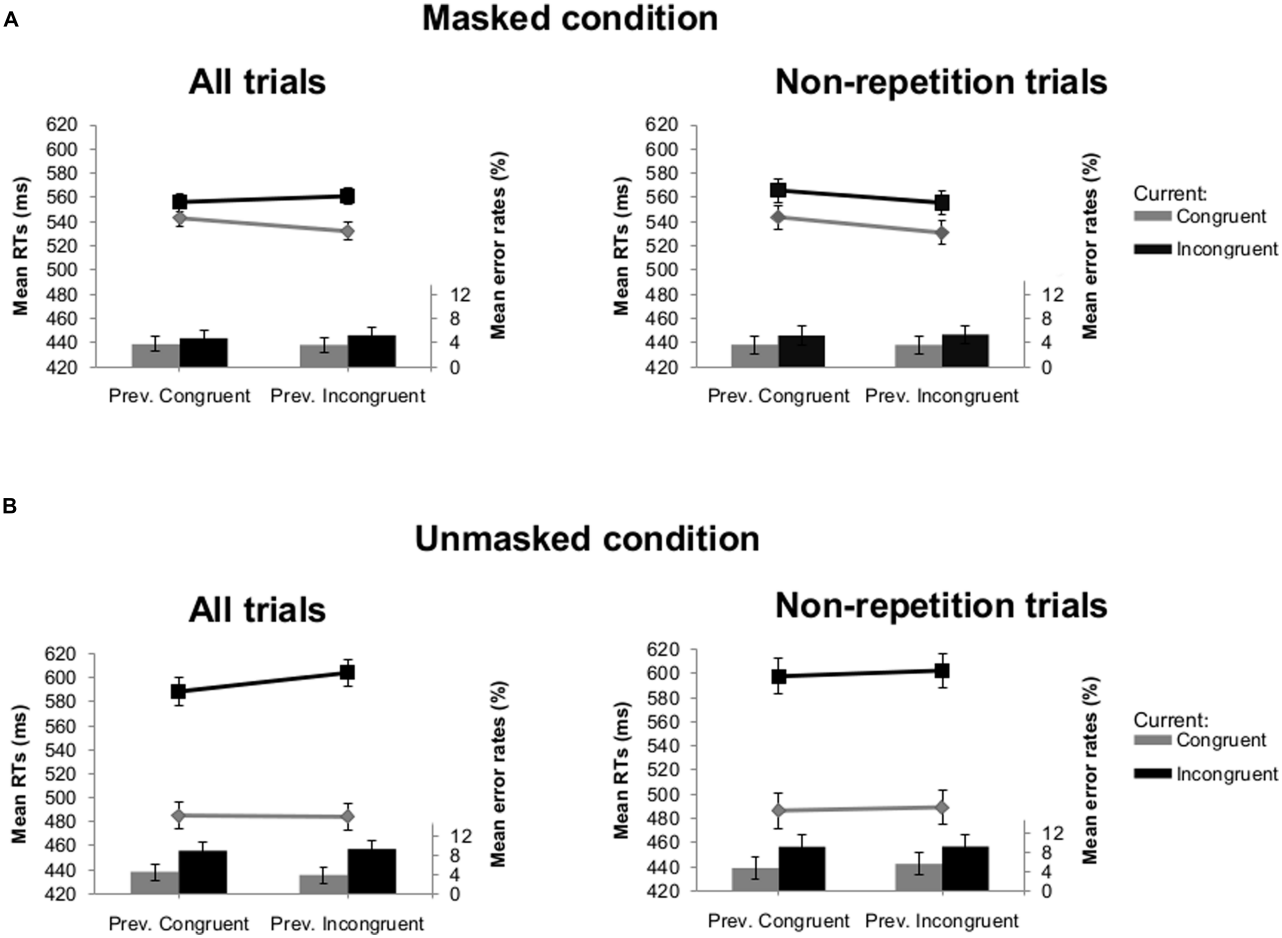
**Mean reaction times (RTs; lines) and error rates (bars) as a function of previous and current congruency in Experiment 1A. (A)** Results of all trials (left) and non-repetition trials (right) in the masked condition. **(B)** Results of all trials (left) and non-repetition trials (right) in the unmasked condition. Error bars reflect 95% within-subject confidence intervals (see Masson and Loftus, 2003).

In *Experiment 1A*, this analysis showed a main effect of current congruency [*F*(1,25) = 196.99, *p* < 0.001] with faster average RTs on congruent (511 ms) compared to incongruent trials (577 ms). There was an interaction between visibility and current congruency [*F*(1,25) = 139.16, *p* < 0.001], indicating larger congruency effects in the unmasked (111 ms) than the masked condition (22 ms). The interaction between visibility and previous congruency was also significant [*F*(1,25) = 4.75, *p* = 0.039]. The difference in RTs after previous congruent and previous incongruent trials was larger in the unmasked (537 ms versus 544 ms) compared with the masked condition (550 ms versus 547 ms). Crucially, there was an interaction between current congruency and previous congruency [*F*(1,25) = 6.78, *p* = 0.015] which was not modulated by visibility (*F* < 1; see **Figure 2**). Follow-up analyses showed that the congruency effect was always *larger* following an incongruent trial compared to a congruent trial [i.e., *irregular* CSE; 29 ms versus 13 ms, *t*(25) = -2.49, *p* = 0.020 in the masked condition; 120 ms versus 103 ms, *t*(25) = -1.73, *p* = 0.097 in the unmasked condition]. To examine the effects of feature repetitions on this unexpected sequential modulation of the congruency effect, we conducted the same analysis after eliminating all trials where the identity of the prime and/or the target of the current trial was the same as the identity of the prime and/or the target of the previous trial (i.e., 43.3% of all trials). We retained on average 90 (SD = 6.7) trials per participant in the masked condition and 88 (SD = 5.7) in the unmasked condition. Importantly, the crucial interaction between current congruency and previous congruency, indicating a CSE, was no longer significant (*F* < 1; see **Figure 2**).

**FIGURE 3:**
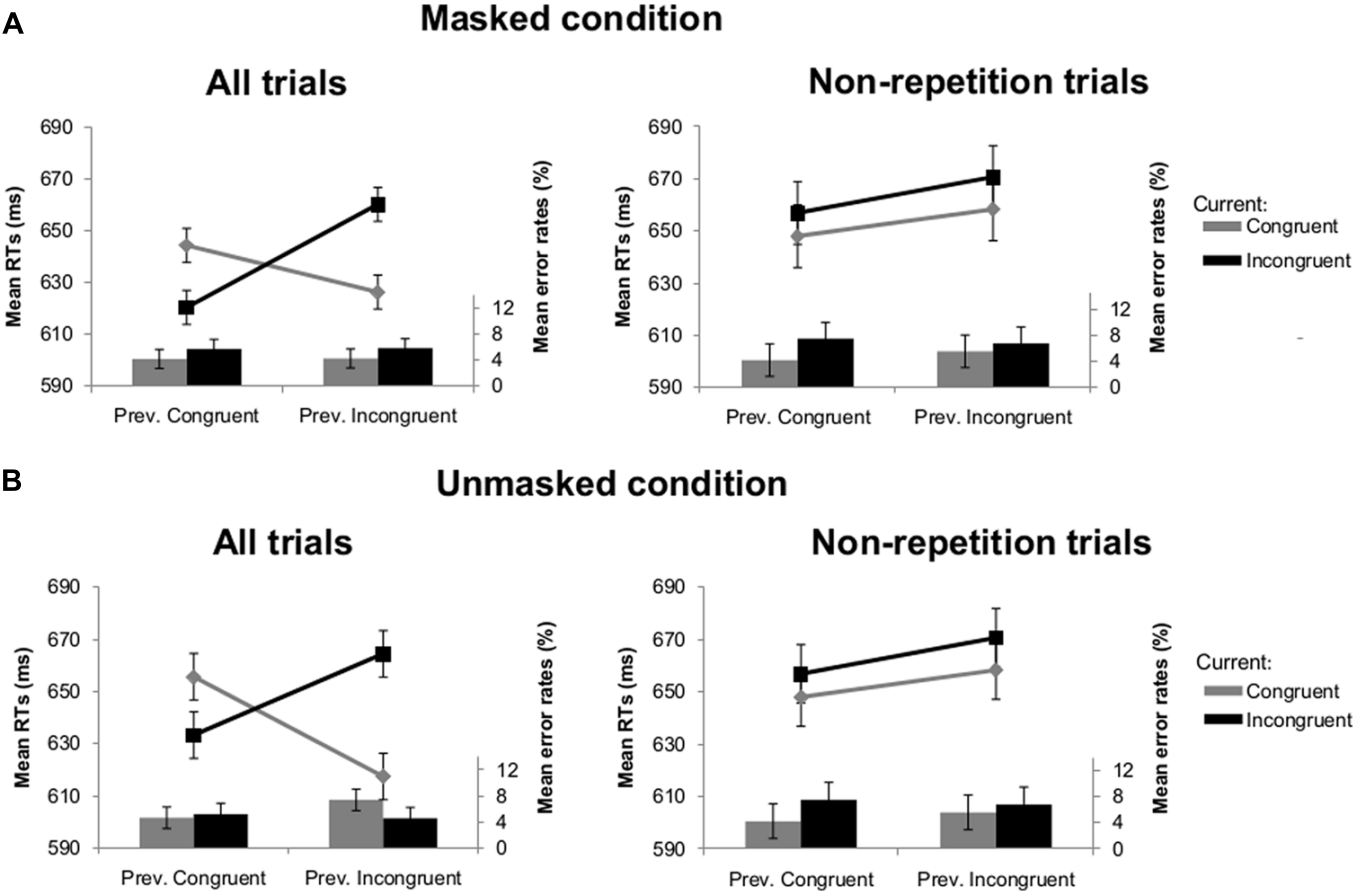
**Mean RTs (lines) and error rates (bars) as a function of previous and current congruency in Experiment 1B. (A)** Results of all trials (left) and non-repetition trials (right) in the masked condition. **(B)** Results of all trials (left) and non-repetition trials (right) in the unmasked condition. Error bars reflect 95% within-subject confidence intervals (see Masson and Loftus, 2003).

The results of *Experiment 1B* were in line with Experiment 1A. A similar main effect of current congruency [*F*(1,26) = 14.28, *p* = 0.001] and interaction between visibility and previous congruency [*F*(1,26) = 10.02, *p* = 0.004] was observed. As in Experiment 1A, the crucial interaction between current congruency and previous congruency was significant [*F*(1,26) = 91.05, *p* < 0.001] and not modulated by visibility (*F* < 1). We again always observed that congruency effects were sharply *enhanced* following incongruent trials compared to congruent trials [34 ms versus -24 ms; *t*(26) = -8.58, *p* < 0.001 in the masked condition; 47 ms versus -22 ms; *t*(26) = -6.20, *p* < 0.001 in the unmasked condition; see **Figure 3**]. After removing all possible repetitions [we retained 85 (SD = 7.3) trials in the masked and 80 (SD = 8.6) trials unmasked condition], the interaction between current congruency and previous congruency again was no longer significant (*F* < 1; see **Figure 3**).

#### Error rates

In *Experiment 1B*, this analysis showed a main effect of visibility [*F*(1,25) = 17.66, *p* < 0.001], with participants making more errors in the unmasked (6.7%) compared to the masked condition (4.3%). We observed a main effect of current congruency [*F*(1,25) = 28.52, *p* < 0.001]: more errors were made on average on incongruent (7.0%) than on congruent (4.0%) trials. There was also an interaction between visibility and current congruency [*F*(1,25) = 10.83, *p* = 0.003], reflecting the fact that the congruency effect was more prominent for the unmasked (4.8%) than for the masked condition (1.3%). Crucially, there was no interaction between current and previous congruency (*F* < 1; see **Figure 2**). None of the other effects reached significance. After removing all possible repetitions, the interaction between current congruency and previous congruency was also not significant (*F* < 1, see **Figure 2**).

For *Experiment 1B* there was an interaction between visibility and current congruency [*F*(1,26) = 14.36, *p* = 0.001]. The congruency effect was larger in the unmasked (4.8%) compared to the masked condition (1.3%). There was no interaction between current and previous congruency [*F*(1,26) = 1.87, *p* = 0.18]. None of the other effects reached significance. After removing all possible repetitions, the analysis showed no interaction between current and previous congruency [*F*(1,26) = 2.19, *p* = 0.15; see **Figure 3**].

#### Prime visibility

Data of the *masked condition* showed that participants correctly categorized primes in 33% of the posttest trials in Experiment 1A and in 38% of the posttest trials in Experiment 1B. This is above chance level performance [i.e., 25%; *t*(25) = 4.78, *p* < 0.001 in Experiment 1A; *t*(26) = 5.99, *p* < 0.001 in Experiment 1B]. Non-significant correlations were found between the individual visibility measure and our index of the CSE (*r* = -0.12, *p* = 0.55 in Experiment 1A; *r* = -0.08, *p* = 0.70 in Experiment 1B). In the *unmasked condition*, the average proportion of correctly categorized primes (77% in both experiments) was clearly above chance level [*t*(25) = 17.34, *p* < 0.001 in Experiment 1A; *t*(26) = 11.84, *p* < 0.001 in Experiment 1B]. Importantly, the visibility in the unmasked condition was significantly higher than in the masked condition [*t*(25) = -14.00, *p* < 0.001 in Experiment 1A; *t*(26) = -8.58, *p* < 0.001 in Experiment 1B].

### DISCUSSION

In Experiment 1 we examined whether the regular CSE (i.e., the Gratton effect) is caused by the monitoring of conflict (Botvinick et al., 2001) or rather by the presence of feature repetitions (Mayr et al., 2003; Hommel et al., 2004). To be able to test both accounts, we used a masked priming paradigm with four stimuli and four responses. By increasing the amount of stimuli and responses, we were able to compare the regular CSE in all trials with the effect in non-repetition trials (i.e., the identity of the prime and/or the target of the previous trial was not repeated on the current trial). Although we expected a regular CSE (i.e., *reduced* congruency effect following an incongruent trial compared to a congruent trial) we observed an opposite pattern in the responses (i.e., *increased* congruency effect following an incongruent trial compared to a congruent trial) when analyzing all trials. This unexpected and irregular CSE cannot be accounted for by the conflict monitoring theory (Botvinick et al., 2001). According to this account, the sequential modulation of the congruency effect is a consequence of an increase in cognitive control following conflict. However, from this perspective it would be hard to explain the current increase of the congruency effect following conflict. Furthermore, this effect disappeared completely when analyzing non-repetition trials only. Thus, as predicted by the feature repetitions account and in line with the results of Mayr et al. (2003), we observed no regular CSE when all feature repetitions were removed. This supports the idea that feature repetitions influence the sequential modulation of the congruency effect. However, given that our full dataset showed an irregular CSE, which is also not predicted by this latter account, the absence of a regular CSE after removing all possible feature repetitions is no convincing evidence in support of the feature repetitions account either. We can only conclude with certainty that feature repetitions have an impact on this irregular CSE. Hence, examining cognitive adaptation with or without feature repetitions might be more difficult to conceive than is often suggested in the literature. Neither the interpretation of the regular CSE in terms of repetition priming effects (Mayr et al., 2003), nor the interpretation in terms of feature integration (Hommel et al., 2004) can explain the irregular CSE that we observed. According to both interpretations, specific feature repetitions lead to a faster reaction on II and CC trials, respectively, compared to CI and IC trials. This difference in RTs is considered to be the underlying source of the *regular* CSE. However, this selective benefit for II and CC trials was not present in our results. In contrast, we found the exact opposite pattern (i.e., slower reaction on II and CC trials, respectively, compared to CI and IC trials). As Mordkoff (2012) pointed out, there are different sorts of repetition trials (i.e., complete repetition, partial repetition in which the relevant or irrelevant information repeats, negative priming repetitions in which the relevant information of the previous trial becomes the relevant information on the current trial) and the removal of all these trials affect the different trial types (i.e., II, CC, IC, CI) at varying degrees. Hence, when repetition trials are included in the design, the proportion of repetition and non-repetition trials differs for each trial type. These varying influences of feature repetitions on each trial type can lead to complex interactions affecting the overall pattern of responses. In conclusion, the observed irregular CSE might be triggered by these complex influences of feature repetitions. Therefore it seems necessary for future studies to investigate the regular CSE in a more ‘clean’ design where feature repetitions are excluded beforehand. Such a straightforward approach seems even more indispensable when considering that studies differ in which specific feature repetitions are removed, making these studies hard to compare (Notebaert and Verguts, 2007). In some recent studies feature repetitions were already excluded *a priori* in the design (Kim and Cho, 2014; Schmidt and Weissman, 2014). We also used this approach in our second experiment in order to investigate whether the regular CSE can be observed when feature repetitions confounds are completely controlled for by excluding them by design.

## EXPERIMENT 2

In our first experiment, we limited the analysis to non-repetition trials to investigate the effects of feature repetitions. Although this is a widely applied approach (e.g., Mayr et al., 2003; Kerns et al., 2004; Ullsperger et al., 2005; Fernandez-Duque and Knight, 2008), it has its limitations. As previously discussed, even though repetition trials are removed from the analysis, the presence of these trials during the experiment could still have an overall influence on the response tendencies of the participants. To preclude every plausible influence of feature repetitions, we set up a second experiment in which all possible repetitions (i.e., prime–prime, target–target, prime–target, target–prime) were excluded beforehand. In Experiment 2 we used the same design as in Experiment 1A, but in this case a trial from one category (e.g., 1–9) was always followed by a trial from the other category (e.g., 2–8). As such, the prime and/or the target were never repeated in two consecutive trials.

### MATERIALS AND METHODS

#### Participants

Twenty-one students (eight females) participated in exchange for course credit. They provided written informed consent before experimentation. One participant was eliminated because the mean error-rate was above 20% and the mean RT was more than two SDs below the average mean. Another participant was eliminated because of a technical failure. Thus, the final sample consisted of 19 participants (seven females), with an age range of 18–25 years (*M* = 20.0, SD = 2.1). All participants had normal or corrected-to-normal vision.

#### Apparatus and stimuli

Apparatus and stimuli were the same as in Experiment 1. The possible prime–target combinations were the same as in Experiment 1A, thus for the congruent and incongruent trials we had two categories of trials (i.e., combinations of 1 and 9 and combinations of 2 and 8). To create neutral trials, the neutral prime “X” was combined with each possible target.

#### Procedure

In general, the same experimental procedure was used as in Experiment 1A (see **Figure 1**). We did change the amount of trials in the different parts of the experiment. In Experiment 2, the participants were presented with eight practice trials, 384 experimental trials and 80 trials in the posttest twice (i.e., once in the masked and once in the unmasked condition). To avoid feature repetitions *a priori*, we also changed the sequence of the trials. A trial of one category was always followed by a trial of the other category. Participants had to respond by pressing the corresponding button on a Cedrus response box (type RB-840).

### RESULTS

RTs above 1000 ms and below 200 ms (2.0% of the data), trials on which an error was made (4.4%) and trials following an error (5.3%) were excluded from further analysis. Mean RTs of correct trials and mean error rates were submitted to a repeated measures ANOVA with the same within-subject factors as in Experiment 1.

#### Reaction times

**FIGURE 4:**
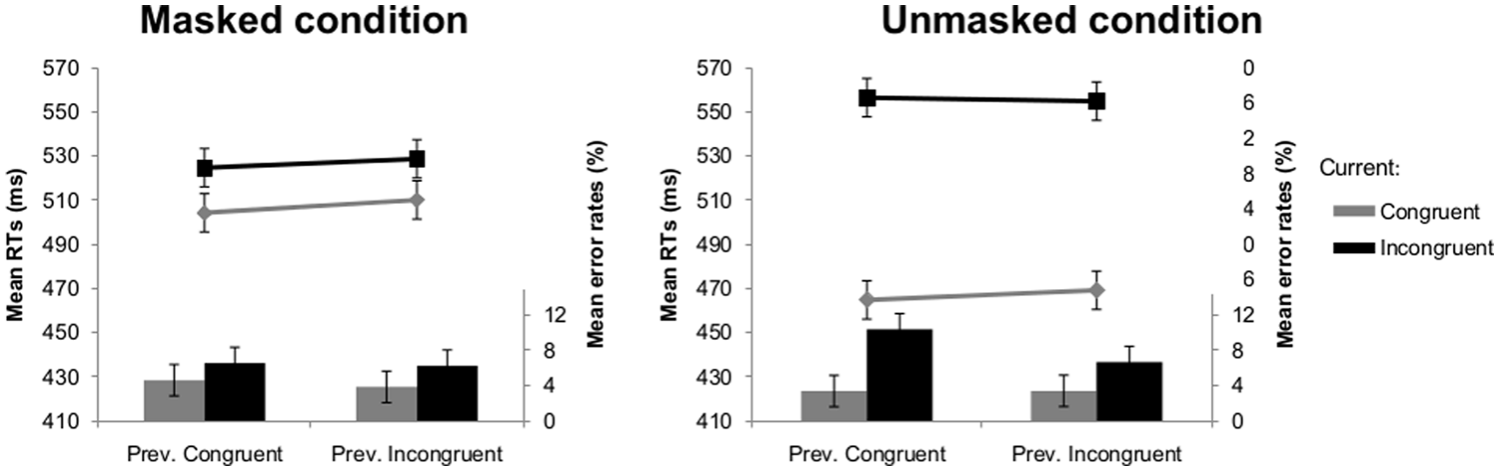
**Mean RTs (lines) and error rates (bars) as a function of previous and current congruency in Experiment 2 in the masked (left) and unmasked (right) condition.** Error bars reflect 95% within-subject confidence intervals (see Masson and Loftus, 2003).

This analysis showed a main effect of current congruency [*F*(1,18) = 69.76, *p* < 0.001], indicating that participants responded slower to incongruent (541 ms) compared to congruent trials (487 ms). The interaction between visibility and current congruency was significant [*F*(1,18) = 67.26, *p* < 0.001]; the congruency effect was smaller in the masked (20 ms) than the unmasked condition (89 ms). Crucially, the interaction between current and previous congruency did not reach significance (*F* < 1), indicating the absence of a regular CSE (see **Figure 4**). None of the other effects reached significance.

#### Error rates

As in the RT-analysis, there was a significant main effect of current congruency [*F*(1,18) = 7.80, *p* = 0.012] with more errors being made on incongruent (7.5%) compared to congruent trials (3.8%). There was also a significant main effect of previous congruency [*F*(1,18) = 4.44, *p* = 0.049]: the error rates on the current trial were higher when the previous trial was congruent (6.2%) compared to incongruent (5.0%). There was a significant interaction between visibility and current congruency [*F*(1,18) = 5.59, *p* = 0.030], reflecting that congruency effects were larger in the unmasked condition (5.1%) than in the masked condition (2.2%). The crucial interaction between current congruency and previous congruency was not significant [*F*(1,18) = 1.80, *p* = 0.197]. However, the three-way interaction was significant [*F*(1,18) = 5.66, *p* = 0.029]. Separate ANOVAs indicated that the interaction between current and previous congruency was not significant in the masked condition (*F* < 1; see **Figure 4**), but was significant in the unmasked condition [*F*(1,18) = 4.83, *p* = 0.041; see **Figure 4**].

#### Prime visibility

To assess prime visibility, we analyzed the average proportion of correctly categorized primes in the posttest. In the *masked condition*, participants correctly categorized primes on 34% of the trials, which is above chance level performance [i.e., 25%; *t*(18) = 2.83, *p* = 0.011]. In the *unmasked condition*, the average proportion of correctly categorized primes (88%) was clearly above chance level [*t*(18) = 19.84, *p* < 0.001]. Importantly, the visibility in the masked condition differed significantly from the visibility in the unmasked condition [*t*(18) = -19.47, *p* < 0.001].

## GENERAL DISCUSSION

In spite of the large amount of research, it is still unclear whether and how cognitive control and consciousness are linked. Many researchers used conflict tasks and analyzed the presence of the regular CSE (i.e., the Gratton effect) in conditions differing in stimulus visibility. However, the results of these studies are not unequivocal (for a review see Desender and Van den Bussche, 2012; Kunde et al., 2012), making it hard to draw strong conclusions on the consciousness-control link. In addition, some researchers argue that the regular CSE does not truly reflect cognitive control, making such conclusions even harder. These researchers suggested that the regular CSE is a consequence of feature repetitions (Mayr et al., 2003; Hommel et al., 2004) rather than cognitive control (Botvinick et al., 2001). We designed three priming experiments to investigate whether the regular CSE is a consequence of cognitive control, and if so, whether this effect also occurs when the conflicting information is masked.

### DOES THE GRATTON EFFECT REFLECT COGNITIVE CONTROL?

When restricting the analysis to non-repetition trials, we observed no regular CSE in Experiment 1. As such, our results seem to corroborate previous findings (Schmidt and De Houwer, 2011; Mordkoff, 2012) in support of the feature repetitions account. However, when all trials were analyzed, we did not observe a regular CSE either but a CSE characterized by an opposite pattern of responses. This makes an interpretation of the regular CSE in terms of feature repetitions not appropriate based on our data. We can only conclude that feature repetitions seem to influence the sequential modulation of the congruency effect in various ways. As discussed before, the *post hoc* removal of feature repetitions is not without problems. To overcome these limitations, feature repetitions were precluded beforehand in some recent studies (Kim and Cho, 2014; Schmidt and Weissman, 2014). In both studies, a reliable regular CSE was found. Like in our study, Schmidt and Weissman (2014) used a priming paradigm, however, with clearly visible primes only. To the best of our knowledge, we are the first to study the regular CSE in both a masked and unmasked condition while controlling for feature repetition confounds. Our analysis of the error rates of Experiment 2 revealed the presence of a regular CSE in the unmasked condition. Although we have to be cautious in interpreting this small effect, it suggests that cognitive control can trigger a regular CSE when the conflicting information is consciously perceived. In contrast to some previous studies (van Gaal et al., 2010; Desender et al., 2013), our study failed to provide evidence for conflict adaptation when the conflicting information is masked. However, it is possible that the difference between both conditions may be caused by a difference in the size of the congruency effect rather than a difference in awareness. In the masked condition the congruency effect was smaller, therefore the amount of conflict might be too small to induce adaptation processes. Furthermore, given that we only observed a small effect, more research is needed to investigate whether our results can be replicated.

Right now, we conclude that the effects of feature repetitions might be more complex than previously suggested and that a ‘clean’ design is needed to examine both competing accounts concerning the regular CSE. Additionally, based on our second experiment we cautiously conclude that there might be some evidence for a regular CSE triggered by cognitive control if the conflicting information is presented clearly visible. This questions the idea that the regular CSE can solely be explained by feature repetitions. Egner (2007) already suggested that neither account can fully explain all the results that are found by different researchers. He points to the possibility of a combination of different processes underlying the regular CSE.

### AN UNEXPECTED FINDING: AN INCREASED CONGRUENCY EFFECT FOLLOWING CONFLICT

In Experiments 1A and 1B, we found an increased congruency effect following an incongruent trial compared to a congruent trial when including all trials in the analysis. As discussed before, we did not anticipate this irregular CSE and neither the conflict monitoring account (Botvinick et al., 2001) nor the feature repetitions accounts (Mayr et al., 2003; Hommel et al., 2004) can fully explain this reversed effect.

Although this irregular CSE seems highly remarkable, a closer look at the literature shows that at least in some studies this effect was also observed (Fischer et al., 2008; Jiang et al., 2013). Fischer et al. (2008) observed a reversed adaptation pattern when comparing adaptation to different kinds of conflict. Furthermore, in a recent study using an affective priming paradigm with masked and unmasked primes, Jiang et al. (2013) compared the regular CSE on response alternation versus response repetition trials. When the response of two consecutive trials was repeated, they observed the same irregular CSE as we did. When the response alternated, they observed a regular CSE (in the conscious condition only). They proposed that the irregular CSE was a consequence of response priming rather than emotional conflict, given that this effect only occurred when analyzing the trials in which the response was repeated. However, as suggested by Mayr et al. (2003), response priming should result in a *regular* CSE, and not a reversed pattern as Jiang et al. (2013) observed. In contrast to the results of Jiang et al. (2013), we observed no regular CSE when analyzing non-repetition trials in the current study. However, we observed an irregular CSE in all trials. Given that response repetition as well as response alternation trials are included in these analysis, this irregular CSE cannot be exclusively attributed to response priming, challenging the conclusion of Jiang et al. (2013). Further research, where stimulus and response repetitions are properly separated, is needed to understand these conflicting results.

Apart from these isolated studies, the irregular CSE that we observed has also been observed in studies in which feature repetitions are avoided by using two different tasks and/or responses between which participants have to switch. For example, Verguts and Notebaert (2008) found evidence for this reversed pattern of responses when the task switches between two trials. In another study participants had to switch between a vertical and horizontal Simon task (Braem et al., 2011). Braem et al. (2011) observed a regular CSE when participants had to respond to both the vertical as well as the horizontal Simon task by pressing buttons with their two hands. However, when they had to respond to one task with their hands and to the other with their feet (i.e., two different response modalities), they observed the same irregular CSE that we did when the response modality switched. The results of both studies are explained by an interpretation of the regular CSE in terms of associative learning (Verguts and Notebaert, 2008, 2009). When participants encounter conflict (i.e., incongruent trial), this is assumed to result in a strengthening of the association between the task-relevant units, which leads to reduced influence of irrelevant information on the following trial (Verguts and Notebaert, 2008, 2009). When stimulus-response associations of task 1 are enhanced as a consequence of conflict, the other stimulus-response associations (including those of task 2) are weakened. Thus, there is less attention for the relevant information of task 2 following an incongruent trial of task 1, leading to the reversed pattern of responses. In Experiment 1A of the current study, a prime from one category (e.g., 1–9) was only combined with a target from the same category. Therefore, the irregular CSE could have been a consequence of a switch between these two categories. We found a reversed pattern of responses when all trials were analyzed and no CSE after removing all feature repetitions. This suggests that task switches only lead to the irregular CSE when feature repetitions are included and that the effect vanishes when repetitions are controlled for. This corroborates the finding of Kim and Cho (2014), who did not observe a regular CSE when the response mode switched (e.g., from right to left hand) in non-repetition trials. However, it may be argued that an interpretation in terms of task switches does not hold for the irregular CSE observed in the current study. First, in Experiment 1B, the different stimulus-options were mixed. In spite of not having two categories we still observed a reversed pattern of responses. Second, it seems unlikely that the participants would categorize the stimuli in these two categories (i.e., 1–9 and 2–8) in the masked condition given that the primes are almost imperceptible. Nevertheless, we did find a reversed effect in this condition. Further research in which the switches between such categories are manipulated is needed to evaluate whether task switches could have had an influence in our studies.

### CONCLUSION

Based on the current results, we conclude that feature repetitions have an influence on the sequential modulation of the congruency effect. However, this influence seems more complex than previously suggested and might not be directly comparable to conflict adaptation effects. Further research is needed to come to a better understanding of the irregular CSE that we observed in our first experiment. Furthermore, to avoid all confounding influences of feature repetitions, it is important to preclude all sorts of feature repetitions in the design to circumvent the shortcomings of *post hoc* removal of repetition trials. To be able to further unravel the influence of cognitive control on the one hand and feature repetitions on the other such designs will be crucial. In our second experiment we used such a design and found limited support for the role of cognitive control in the regular CSE. This was only the case when the irrelevant information was presented clearly visible. However, more research in which feature repetitions are precluded in both an unmasked as well as masked condition is needed to verify whether our results can be replicated.

## Conflict of Interest Statement

The Guest Associate Editor James R. Schmidt, declares that, despite being affiliated to the same institution as author Elke Van Lierde, the review process was handled objectively and no conflict of interest exists. The authors declare that the research was conducted in the absence of any commercial or financial relationships that could be construed as a potential conflict of interest.

## Footnotes

1 This was done to avoid contingency biases. If we would fully cross all possible stimulus combinations, each prime could be combined with three different incongruent targets and only one congruent target. When in addition the congruent and incongruent trials are balanced (i.e., each 50%) in the design, the primes are more often combined with a congruent target than can be expected by chance (i.e., 25% in our design), leading to higher contingency between this prime and target (Schmidt and De Houwer, 2011; Mordkoff, 2012). If participants learn these contingencies, this can lead to faster and more correct responses on high contingency trials relative to low contingency trials (i.e., contingency effect; Schmidt et al., 2007). Moreover, this contingency effect is smaller when the previous trial is a low contingency (or incongruent) trial compared to a high contingency (or congruent) trial (Schmidt et al., 2007). Thus, when contingency biases are not controlled for, the regular CSE could reflect a sequential modulation of the contingency effect.

2 We initially included neutral trials in the experiments to be able to investigate whether the regular CSE was caused by previous incongruent trials, previous congruent trials or both (Desender et al., 2013). Because of the unexpected findings reported below, these analyses were no longer useful. Therefore, we report only the results of the analysis on congruent and incongruent trials. Note, however, that conceptually the same results were obtained when including neutral trials in the analysis.
